# Relationship between the severity of functional mitral regurgitation at admission and one-year outcomes in patients hospitalized for acute heart failure with mildly reduced ejection fraction

**DOI:** 10.1186/s12872-024-04017-4

**Published:** 2024-07-13

**Authors:** Zhenhua Wang, Yue Zeng, Hanzhao Qiu, Li Chen, Jun Chen, Chaosheng Li

**Affiliations:** 1 Department of Cardiology, The Second Affiliated Hospital of Shenzhen University (The People’s Hospital of Baoan Shenzhen), Shenzhen, 518100 China; 2 Graduate School, Guangdong Medical University, Zhanjiang, 524000 China; 3 Department of Cardiology, Baoan District Central Hospital of Shenzhen, Shenzhen, 518100 China

**Keywords:** Functional mitral regurgitation, Heart failure with mildly reduced ejection fraction, Left ventricular ejection fraction

## Abstract

**Background:**

The epidemiological distribution of functional mitral regurgitation (FMR) in heart failure (HF) and mildly reduced ejection fraction (HFmrEF) patients and its impact on outcomes remains unclear. We attempt to investigate the prognosis of FMR in patients with HFmrEF.

**Methods:**

The HF center registry study is a prospective, single, observational study conducted at the Second Affiliated Hospital of Shenzhen University, where 2330 patients with acute HF (AHF) were enrolled and 890 HFmrEF patients were included in the analysis. The patients were stratified into three categories based on the severity of FMR: none/mild, moderate, and moderate-to-severe/severe groups. Subsequently, a comparison of the clinical characteristics among these groups was conducted, along with an assessment of the incidence of the primary endpoint (comprising all-cause mortality and readmission for HF) during a one-year follow-up period.

**Results:**

The one-year follow-up results indicated that the primary composite endpoint occurrence rates in the three groups were 23.5%, 32.9%, and 36.5%, respectively. The all-cause mortality rates in the three groups were 9.3%, 13.7%, and 16.4% respectively. Survival analysis demonstrated a statistically significant difference in the occurrence rates of the primary composite endpoint and all-cause mortality among the three groups (*P* < 0.05). Multifactor Cox regression revealed that moderate FMR and moderate-to-severe/severe FMR were independent risk factors for adverse clinical prognosis in HFmrEF patients, with hazard ratios and 95% confidence intervals of 1.382 (1.020–1.872, *P* = 0.037) and 1.546 (1.092–2.190, *P* = 0.014) respectively.

**Conclusions:**

Moderate FMR and moderate-to-severe/severe FMR independently predict an unfavorable prognosis in patients with HFmrEF.

## Introduction

Heart failure (HF) represents a significant global public health concern, impacting a staggering population of over 23 million individuals worldwide [1]. HF with mid-range ejection fraction (HFmrEF) was introduced by the 2016 European Society of Cardiology (ESC) HF guidelines and 2018 Chinese HF guidelines in an attempt to address the “gray zone” with HF of left ventricular ejection fraction (LVEF) between 40% and 49% [2, 3]. Within the 2021 ESC guidelines for diagnosing and treating HF, a redefinition of HFmrEF occurred. It now stands for HF with mildly reduced ejection fraction (HFmrEF), a terminology that encourages further investigation and research in this particular ejection fraction (EF) range. This adjustment was made due to the comparatively limited amount of research focused on HFmrEF compared to HF with reduced ejection fraction (HFrEF) and HF with preserved ejection fraction (HFpEF). By stimulating research within the HFmrEF EF range, a deeper understanding and more targeted approaches can be developed for the care and management of these patients. [4] Functional mitral regurgitation (FMR) is a common complication of HF and is associated with adverse outcomes in patients with HFrEF. [5–8] Given the limited domestic and international research on the relationship between FMR and HFmrEF patients, the epidemiological distribution of FMR in HFmrEF patients and its impact on outcomes remains unclear. Consequently, this study aims to investigate the epidemiological characteristics of FMR in Chinese patients hospitalized for acute heart failure (AHF) with mildly reduced ejection fraction and its influence on patient prognosis.

## Methods

### Study design and data collection

HF center registry study is a prospective, observational study conducted at the Second Affiliated Hospital of Shenzhen University, focusing on patients diagnosed with AHF. It has been registered in the Chinese clinical trial registry (ChiCTR1800017226). Patients with AHF were recruited during their initial admission and followed by an attending physician after discharge. Baseline data were collected, including demographic information, medical history, vital signs, laboratory test results, echocardiographic parameters, and medication usage. The treatment of HF is not fixed and is set by the attending physicians. This study evaluated the clinical features and prognostic risk factors of patients with AHF. Ethical guidelines outlined in the Declaration of Helsinki were strictly adhered to, and the study protocol received approval from the hospital’s Ethics Committee. Furthermore, all patients provided written informed consent before participating in the study.

### Patients and definitions

Patients aged ≥ 18 years with HFmrEF were included in the study and diagnosed according to the 2021 guidelines set by the ESC for acute and chronic HF. [4] Echocardiography confirmed LVEF between 41% and 49%; New York Heart Association (NYHA) functional class II-IV. Exclusion criteria comprised organic valvular heart disease, congenital heart disease, heart transplantation, renal replacement therapy, acute coronary syndrome, or coronary artery revascularization during hospitalization to avoid the inclusion of acute ischemic mitral regurgitation.

### Echocardiographic assessment of FMR

An echocardiogram was conducted within 72 h after admission. The degree of FMR severity was assessed using a blend of qualitative, semiquantitative, and quantitative echocardiographic parameters and categorized from grade 0 to grade 4 + by the guidelines set forth by the American Society of Echocardiography [9]. The research was classified into three classifications: none (grade 0)/mild (grade 1+), moderate (grade 2+), and moderate-to-severe/severe FMR (grade 3 + and 4+). Atrial and ventricular dimensions were evaluated using the M-mode technique. LVEF was evaluated using the biplane Simpson method. Systolic pulmonary artery pressure (SPAP) was estimated by quantifying the pressure difference caused by regurgitation of the tricuspid valve.

### Outcomes

The primary composite endpoint was defined as all-cause mortality and/or HF readmissions 1 year later, with all-cause mortality as the secondary endpoint. Patients who died during hospitalization, were lost to follow-up, or received mitral valve repair (MVR) during hospitalization and follow-up were excluded from the event analysis. The determination of endpoints was conducted by qualified cardiologists who were blinded to the clinical study.

### Statistical analysis

Statistical analysis was performed using SPSS 26.0 software, and graphical representations were generated using GraphPad Prism 8.0. Continuous data are displayed as median, and the Kruskal‑Wallis H-rank test was used to compare them. Categorical data are expressed as percentages and χ^2^ tests were used for comparison. The Kaplan-Meier method was employed to illustrate the cumulative probability of events, while the log-rank test was used to compare the overall survival rates among different groups. The association between the candidate variables and the primary or secondary endpoints was examined using the Cox proportional hazards model. Multivariate analysis included variables identified as predictors of the outcome in the univariate analysis (*P* < 0.2). Additionally, factors known to influence the outcomes and factors accounting for multicollinearity were also considered. The variables included in the final model comprised age, loop diuretic usage, beta-blocker usage, spironolactone usage, serum sodium levels, creatinine levels, N-terminal-B-type natriuretic peptide (NT-pro-BNP) levels, NYHA functional class, presence of diabetes, and atrial fibrillation. Statistical significance was defined as a *P*-value < 0.05.

## Results

**Fig. 1:**
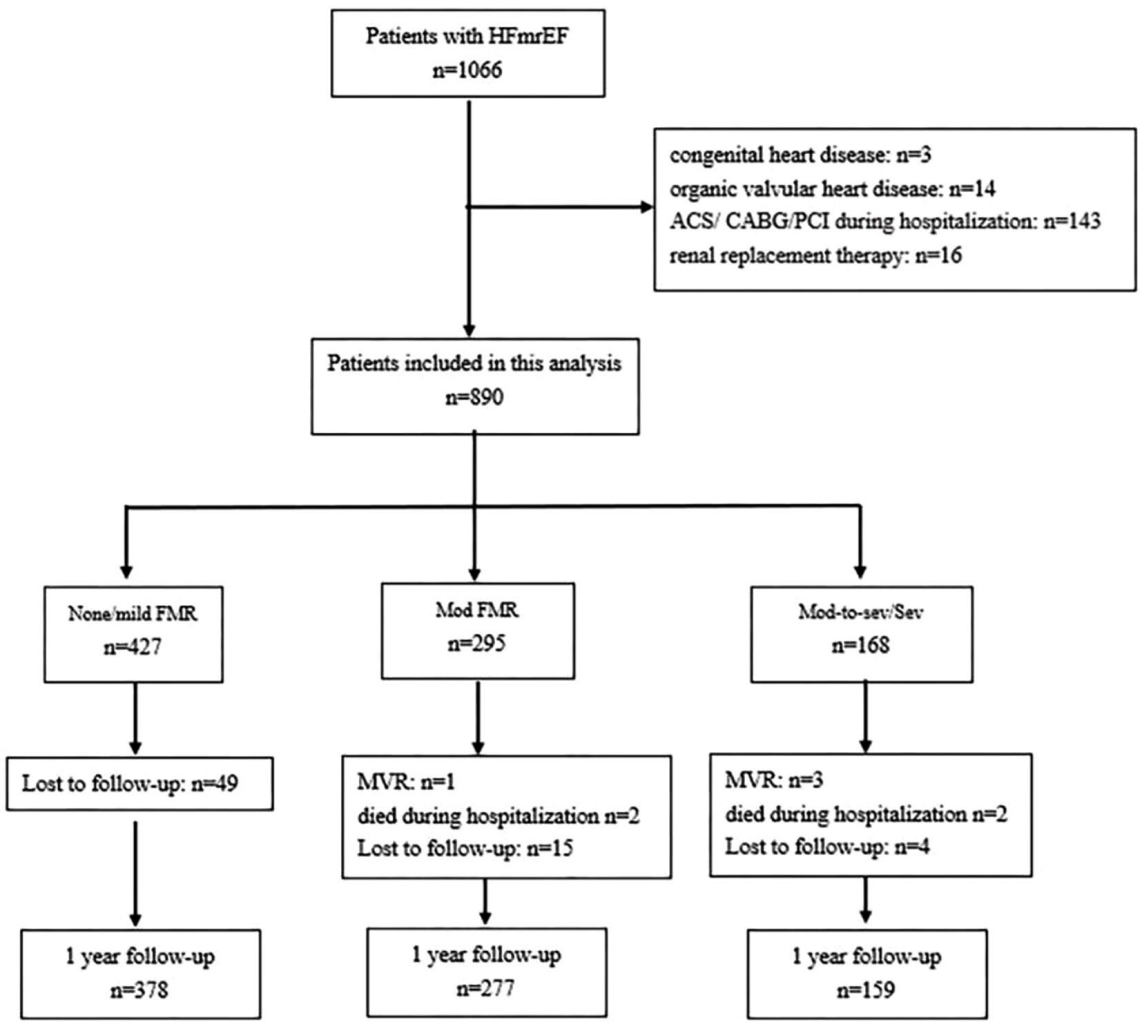
Flow chart depicting study cohort selection. HFmrEF, heart failure with mildly reduced ejection fraction; FMR, functional mitral regurgitation; ACS, acute coronary syndrome; CABG, coronary artery bypass graft; MVR, mitral valve repair; PCI, percutaneous coronary intervention

The disposition of the study is shown in Fig. 1. 176 patients were excluded based on the study exclusion criteria. 890 patients were included in the study cohort. According to the severity of FMR, there were 427 cases (48.0%) in the none/mild FMR group, 295 cases (33.1%) in the moderate FMR group, and 168 cases (18.9%) in the moderate-to-severe/severe FMR group.

### Baseline characteristics

**Fig. 2:**
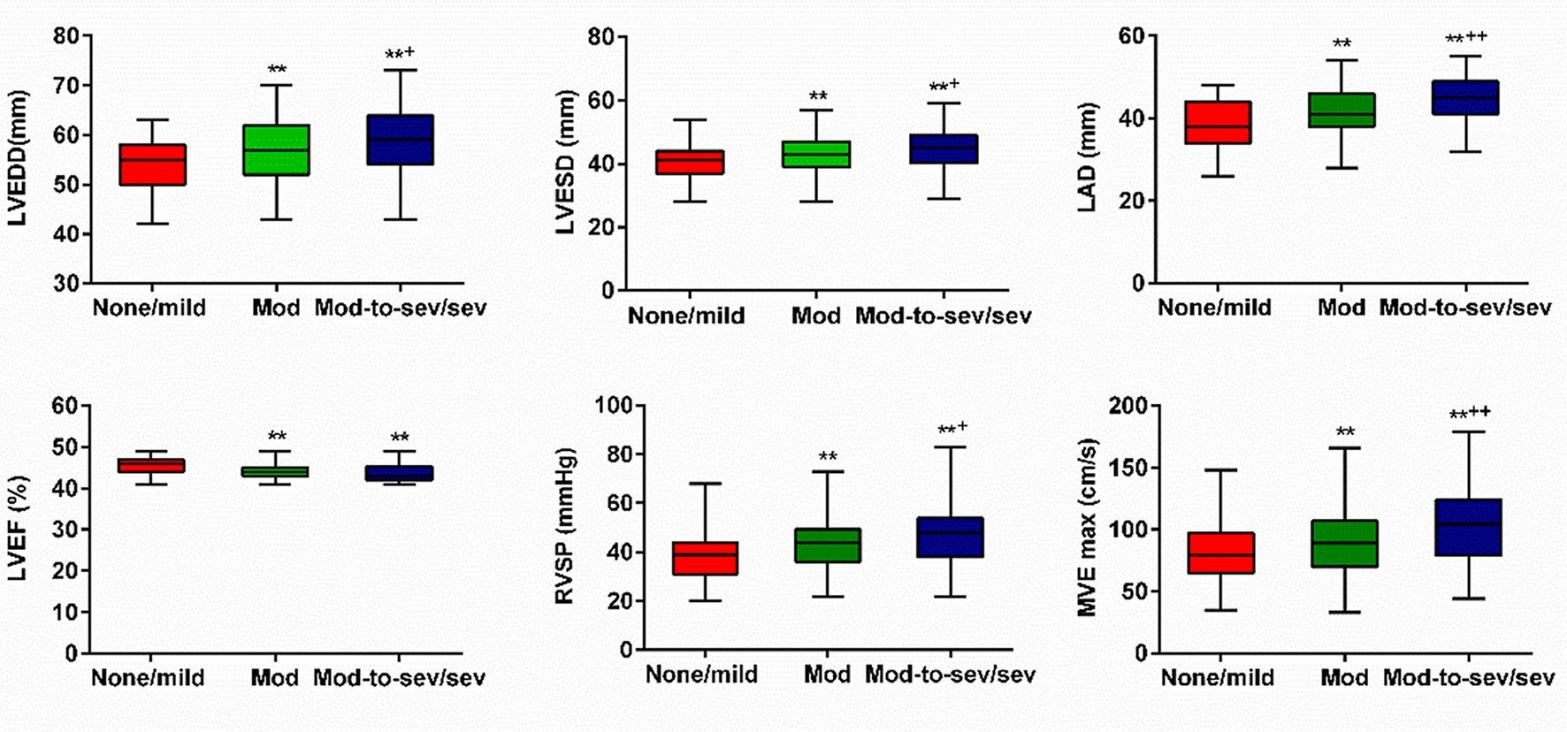
Comparison of echocardiographic parameters of HFmrEF patients in the 3 Groups of FMR. LVEDD, left ventricular end-diastolic diameter; LVESD, left ventricular end-systolic diameter; LAD, left atrial diameter; LVEF, left ventricular ejection fraction; RVSP, right ventricular systolic pressure; MVE max, mitral value maximum E velocity; Mod, moderate; Mod-to-sev/sev, moderate-to-severe/ severe; *P<0.05 and **P<0.01, compared with the group of none/mild FMR; ^+^*P*<0.05 and ^++^*P*<0.01, compared with the group of moderate FMR

The foundational attributes of the three cohorts are presented in Table 1, while the post hoc pairwise assessments for echocardiographic information are visualized in Fig. 2. Individuals with a heightened degree of FMR displayed a greater trend towards advanced age, the presence of atrial fibrillation, classification within NYHA cardiac function class III or IV, as well as a higher propensity for reduced mean arterial pressure and elevated NT-pro-BNP levels, when contrasted with those exhibiting none/mild FMR (*P* < 0.05). Patients presenting with moderate or moderate-to-severe/severe FMR exhibited an enlarged left atrial diameter (LAD), elevated SPAP, increased left ventricular end-diastolic diameter (LVEDD), expanded left ventricular end-systolic diameter (LVESD), and a higher mitral valve flow rate (*P* < 0.05). Furthermore, they manifested a reduced LVEF compared to individuals with none or mild FMR (*P* < 0.05).

**Fig. 3:**
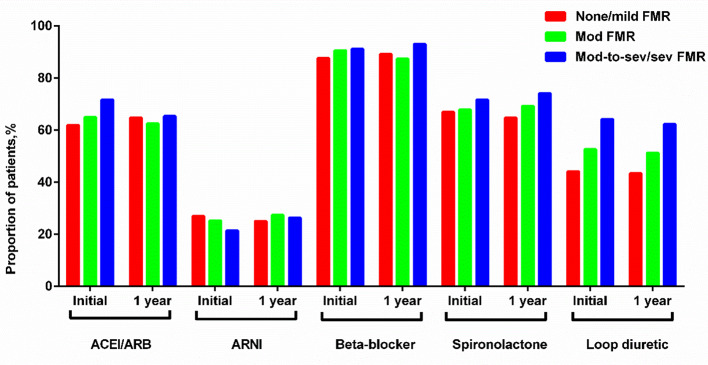
Drug treatments of HFmrEF patients at entry and 1 year later. ACEI, angiotensin-converting enzyme inhibitor; ARB, angiotensin receptor blocker; FMR, functional mitral regurgitation; ARNI, angiotensin receptor neprilysin inhibitor

Figure 3 illustrates the status of guideline-directed medical therapy (GDMT) in HFmrEF patients at discharge and one-year follow-up. Among HFmrEF patients with moderate-to-severe/severe FMR, 71.7% used angiotensin-converting enzyme inhibitor (ACEI) / angiotensin receptor blocker (ARB), 21.4% used angiotensin receptor neprilysin inhibitor (ARNI), 91.2% used beta-blockers, 71.7% used spironolactone, and 64.2% used diuretics. In the none /mild FMR and moderate FMR groups, the utilization rates of ACEI/ARB, spironolactone, ARNI, and beta-receptor blockade were similar to those in the moderate-to-severe/severe FMR group. However, the use of diuretics was higher in HFmrEF patients with moderate-to-severe/severe FMR. There was no significant change in the utilization of GDMT in each group during the 1-year follow-up period.

**Table 1:** Baseline characteristics of patients stratified according to FMR

Variables	All (*n* = 890)	None/mild FMR (*n* = 427)	Mod FMR (*n* = 295)	Mod-to-sev/sev FMR(*n* = 168)	*P*-value
Age, years	64 (53, 75)	61 (50, 71)	66 (57, 76)	66 (54, 78)	<0.001
Female, n (%)	246(27.6%)	106(24.8%)	85 (28.8%)	55(32.7%)	0.130
Mean arterial pressure, mmHg (IQR)	99.3(88.3, 111.0)	100.0(89.7, 111.7)	100.3 (88.7, 112.0)	95.5 (84.4, 108.2)	0.026
Heart rate, b.p.m (IQR)	74 (66. 90)	73 (66, 90)	74 (66, 92)	74(66, 88)	0.474
BMI, kg/m^2^ (IQR)	23.3(21.3, 26.2)	23.4 (21.5, 25.9)	23.1(20.9, 26.2)	23.5 (21.5, 26.4)	0.481
Aetiology, n (%)					
Ischaemic	633 (71.1%)	310(72.6%)	212 (71.9%)	111(66.1%)	0.270
Idiopathic dilated	84(9.4%)	31 (7.3%)	31 (10.5%)	22 (13.1%)	0.067
Medical history, n (%)					
Hypertension	453 (50.9%)	213(49.2%)	166(56.3%)	77(45.8%)	0.060
Diabetes	260 (29.2%)	119 (27.9%)	97 (32.9%)	44(26.2%)	0.219
Chronic kidney disease	191 (21.5%)	88(20.6%)	70 (23.7%)	33 (19.6%)	0.493
Stroke	77 (8.7%)	39 (9.1%)	24 (8.1%)	14 (8.3%)	0.884
Atrial fibrillation	251 (28.2%)	84(19.7%)	96 (32.5%)	71 (42.3%)	<0.001
NYHA functional class 0.001					
II	193 (21.7%)	112 (26.2%)	60 (20.3%)	21 (12.5%)	
III	468 (52.6%)	226 (52.9%)	152 (51.5%)	90 (53.6%)	
IV	229 (25.7%)	89 (20.8%)	83 (28.1%)	57 (33.9%)	
Laboratory inspection					
N-terminal-B-type natriuretic peptide, pg/mL (IQR)	778.2(298.7, 1870.0)	469.2(220.0, 1098.0)	968.3(387.1, 2279.0)	1489.0(664.9, 2852.0)	<0.001
Creatinine, µmol/L (IQR)	87.0 (72.0, 110.3)	86.3 (71.9, 107.8)	88.0 (71.0, 112.0)	90.2 (74.9, 114.2)	0.221
Serum sodium, mmol/L (IQR)	138.4(136.1, 140.8)	138.5(136.6, 140.8)	138.1(136.0, 140.8)	138.6(136.1, 141.0)	0.564
Echocardiographic findings					
Left ventricular end-diastolic diameter, cm (IQR)	5.6 (5.2,6.1)	5.5 (5.0, 5.8)	5.7 (5.2, 6.2)	5.9 (5.4, 6.4)	<0.001
Left ventricular end-systolic diameter, cm (IQR)	4.2 (3.8,4.7)	4.1 (3.7, 4.4)	4.3 (3.9, 4.7)	4.5 (4.0, 4.9)	<0.001
Left atrial diameter, cm (IQR)	4.1 (3.6, 4.6)	3.8 (3.4, 4.4)	4.1 (3.8, 4.6)	4.5 (4.1, 4.9)	<0.001
Left ventricular ejection fraction, %(IQR)	45.0 (43.0,46.0)	46.0 (44.0, 47.0)	44 0.0(43.0, 45.0)	43.0 (42.0, 45.4)	<0.001
Mitral value maximum E velocity, cm/s (IQR)	87.0(68.0, 107.0)	79.0 (65.0, 97.0)	89.0 (70.0, 107.0)	104.5 (79.0,124.0)	<0.001
Right ventricular systolic pressure, mmHg (IQR)	43.0 (35.0, 50.0)	39.0 (31.0, 44.0)	44.0 (36.0, 49.3)	48.0 (38.3, 54.0)	<0.001

FMR, functional mitral regurgitation; IQR, interquartile range; BMI, body mass index; NYHA, new york heart association

### Post-discharge outcomes

**Fig. 4:**
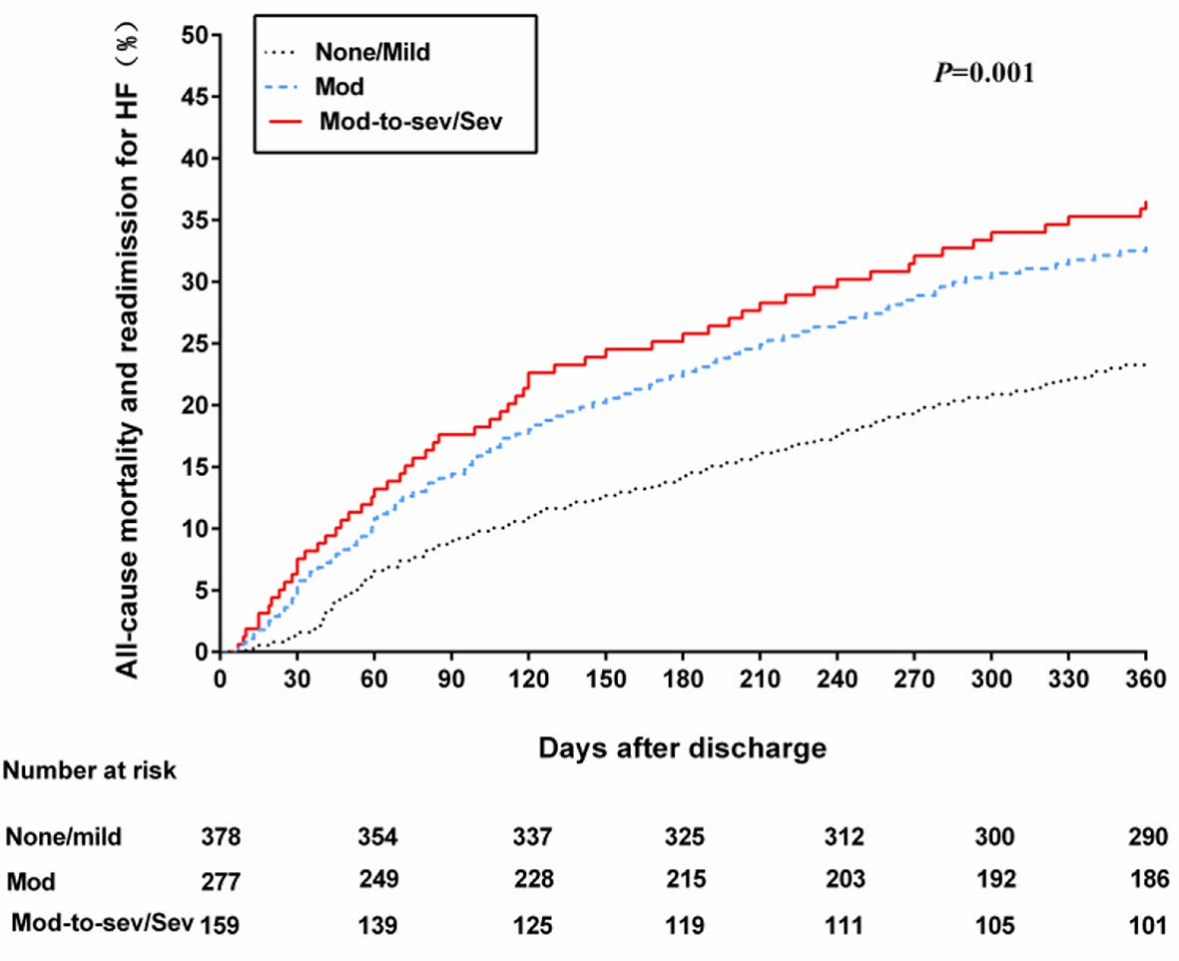
Kaplan-Meier curve estimates of the all-cause mortality and readmission for HF in the 3 Groups of FMR

Approximately 29.2% of the patients experienced the combined outcome of all-cause mortality and readmission for HF during the one-year follow-up period. Among patients classified with none/mild FMR, the rate of the composite endpoint was 23.5%. In patients with moderate FMR, the rate increased to 32.9%, while patients with moderate-to-severe/severe FMR exhibited the highest rate at 36.5%. These findings were supported by an unadjusted log-rank test for trends (*P* = 0.001) (Fig. 4). Based on univariate analysis, it was observed that patients diagnosed with moderate or moderate-to-severe/severe FMR exhibited a heightened likelihood of reaching the composite endpoint compared to individuals with none or mild FMR (Table 2). Moreover, in the multivariate analysis, patients with moderate FMR or moderate-to-severe/severe FMR continued to demonstrate a significantly elevated risk of the composite endpoint when compared to patients with none or mild FMR [hazard ratio (HR) of 1.382, 95% confidence interval (CI) of 1.020–1.872, *P* = 0.037 for moderate FMR; HR of 1.546, 95% CI of 1.092–2.190, *P* = 0.014 for moderate-to-severe/severe FMR].

**Table 2:** COX regression analysis of all-cause mortality and readmission for HF in patients with HFmrEF stratified by FMR

FMR	No. of patients	No. of patients with events (%)	Unadjusted HR	95%CI	*P*-value	Adjusted HR	95%CI	*P*-value
None/mild	378	89 (23.5%)	1.000			1.000		
Mod	277	91(32.9%)	1.508	1.126–2.020	0.006	1.382	1.020–1.872	0.037
Mod-to-sev/Sev	159	58(36.5%)	1.733	1.245–2.412	0.001	1.546	1.092–2.190	0.014

Adjusted for age, loop diuretic, beta-blocker, spironolactone, serum sodium, creatinine, N-terminal-B-type natriuretic peptide, NYHA functional class, diabetes, atrial fibrillation

**Fig. 5:**
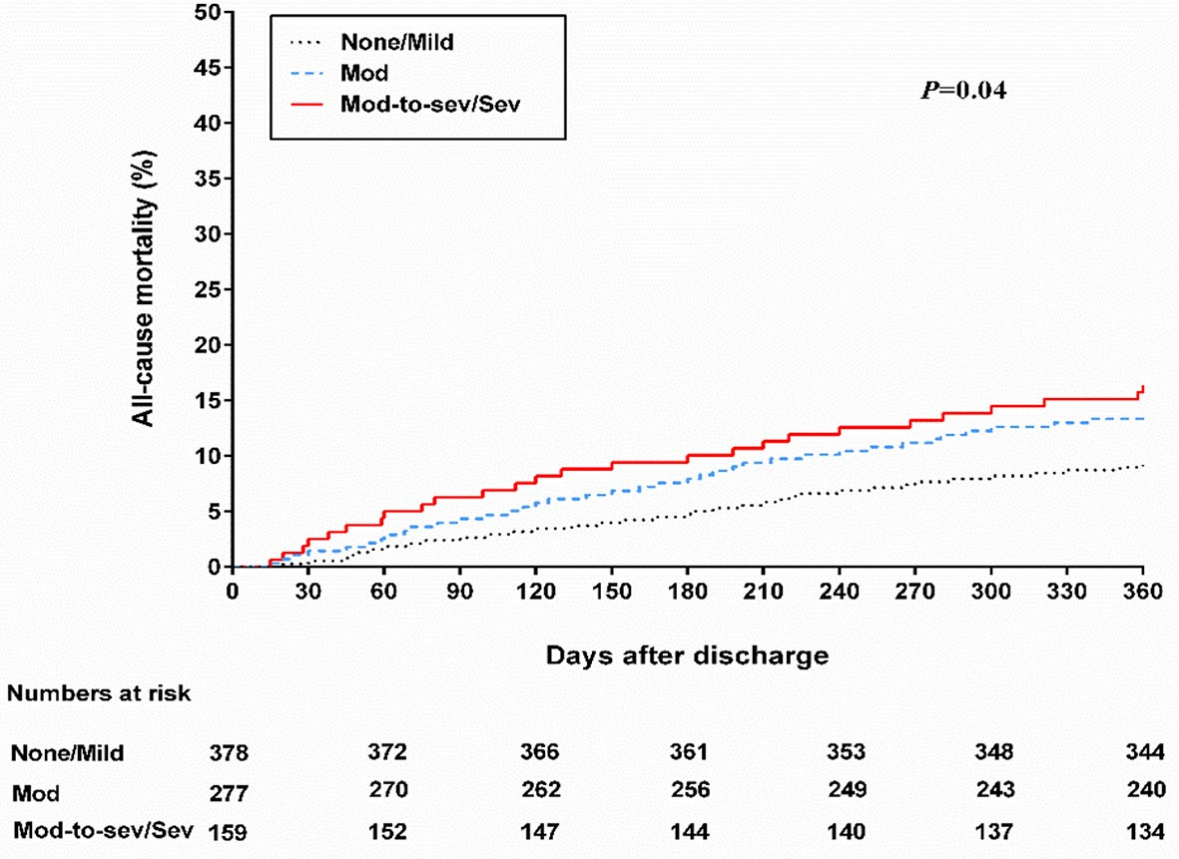
Kaplan-Meier curve estimates of the all-cause mortality in the 3 Groups of FMR

The one-year all-cause mortality rate was 12.2% among patients diagnosed with HFmrEF. Comparatively, patients with none/mild FMR had a one-year all-cause mortality rate of 9.3%, while those with moderate FMR had a rate of 13.7%. Patients with moderate-to-severe/severe FMR had a slightly higher rate of 16.4%. These statistics were obtained through an unadjusted log-rank test for trends, which yielded a *P*-value of 0.04 (Fig. 5). Through both univariate and multivariate analysis (Table 3), it was found that there was no notable increase in the risk of all-cause mortality for patients with moderate FMR when compared to patients with none/mild FMR. However, the univariate analysis did identify a significant elevation in the risk of all-cause mortality for patients with moderate-to-severe/severe FMR. The multivariate analysis further indicated that patients with moderate-to-severe/severe FMR had a somewhat higher risk of all-cause mortality compared to those with none/mild FMR. Still, the difference did not reach statistical significance.

**Table 3:** COX regression analysis of all-cause mortality for HF in patients with HFmrEF stratified by functional mitral regurgitation

FMR	No. of patients	No. of patients with death (%)	Unadjusted HR	95%CI	*P*-value	Adjusted HR	95%CI	*P*-value
None/mild	378	35 (9.3%)	1.000			1.000		
Mod	277	38(13.7%)	1.523	0.962–2.410	0.073	1.438	0.895–2.308	0.133
Mod-to-sev/Sev	159	26(16.4%)	1.851	1.114–3.074	0.017	1.632	0.958–2.781	0.071

Adjusted for age, gender, beta-blocker, loop diuretic, creatinine, NYHA functional class, chronic kidney disease, atrial fibrillation, N-terminal-B-type natriuretic peptide

## Discussion

In our study focused on patients with HFmrEF, we investigated the occurrence and prognostic significance of different degrees of FMR. Our main findings can be summarized as follows: Firstly, we observed that the prevalence of moderate or moderate-to-severe/severe FMR was 52.0%; secondly, moderate and moderate-to-severe/severe FMR was associated with an increased risk of the composite endpoint (all-cause mortality and readmission for HF); thirdly, the risk of all-cause death was not significantly increased in HFmrEF patients with moderate or moderate-to-severe/severe FMR.

FMR is a prevalent condition among individuals affected by chronic HF. It manifests in patients without structural abnormalities in the mitral valve leaflets and is primarily attributed to the remodeling of the left ventricle [10]. The previous report has shown that moderate to severe FMR occurred in approximately 53% of patients with HFrEF. [11] Another study showed that 38% of patients with HFpEF have moderate to severe FMR, while in patients with HFmrEF, the prevalence of moderate to severe FMR is as high as 54%, in HFrEF patients, this percentage increases to 64% [12]. In this study, 52.0% of patients with HFmrEF were found to have moderate to severe FMR. These findings suggest that HFmrEF patients often exhibit concurrent moderate to severe FMR, demonstrating a higher similarity to HFrEF patients regarding FMR epidemiology.

FMR in individuals with HF has been correlated with unfavorable prognostic outcomes. [13]. The ATTEND study [8] revealed that moderate or severe FMR increases the risk of readmission for HF or all-cause mortality by 41% in HFrEF compared with the absence of FMR. Furthermore, in the HFpEF group, patients exhibiting mild or moderate/severe FMR face an elevated risk of reaching the composite endpoint compared to individuals without FMR. An additional study examined FMR’s influence on short-term detrimental events among hospitalized patients experiencing acute HF. The findings revealed a corresponding 50% and 60% increase in the risk of composite endpoint events within 90 days for patients with moderate and severe FMR, respectively, compared to those with none or mild FMR, specifically in individuals diagnosed with HFrEF. [14] We found that the risk of developing endpoint events in HFmrEF patients with moderate FMR and moderate-severe/severe FMR increased by 37% and 52% respectively, and moderate and moderate-severe/severe FMR could predict adverse prognosis in HFmrEF patients. We also found that moderate and moderate-severe/severe FMR did not significantly increase the risk of all-cause mortality within one year in HFmrEF patients. However, Georg et al. suggested that severe FMR increases the risk of death in HFrEF patients by 76% during a median follow-up of 62 months. [7] In summary, the short-term prognosis of FMR in HFmrEF patients is similar to that of HFrEF. There are several reasons for this finding. First, the guidelines gradually provide more detailed and strengthened GDMT for HFmrEF patients. Secondly, increasing post-hoc analysis of randomized controlled trials suggests that drugs may be as effective in patients with HFmrEF as in patients with HFrEF. The pathophysiological characteristics of HFmrEF are more similar to HFrEF. [15–18]

The mechanisms of FMR are as follows [19]: left atrial and ventricular enlargement leading to mitral annular dilation; left ventricular remodeling and morphological changes resulting in disorganized papillary muscle arrangement and functional abnormalities; asynchronous contraction of the myocardium. Simultaneously, FMR induces elevated left atrial pressure and volume, contributing to the progressive dilation of the left ventricle and left atrium. This exacerbates FMR and perpetuates a cyclic pattern of worsening left ventricular dilation and dysfunction. The baseline data of this study showed that patients with moderate to severe FMR had larger NT-proBNP and left heart structures, as well as lower LVEF, indicating a more severe condition.

Guidelines emphasize the importance of early initiation of optimal medical therapy (OMT) for pre-discharge and early post-discharge follow-up of patients hospitalized for AHF. Identifying early indicators that reflect signs, symptoms, and volume status in AHF patients is crucial. AHF patients often experience worsening FMR, a dynamic condition influenced by left atrial pressure and left ventricular volume loading status. The prognostic significance of FMR severity on acute decompensation and after hemodynamic stabilization in AHF patients remains uncertain. Determining the appropriate timing to evaluate FMR for more aggressive treatment remains unclear. Studies on HFrEF have demonstrated that FMR severity after hemodynamic stabilization is linked to short-term and long-term prognosis in AHF patients. [8, 14, 20] Other studies have indicated that FMR severity on admission is associated with poorer post-discharge outcomes in AHF patients. [11, 21] This study highlights that moderate or moderate-to-severe/severe FMR on admission independently predicts an unfavorable prognosis in patients with HFmrEF. Evaluating FMR on admission for AHF could provide a valuable opportunity for early identification and characterization of patients, enabling timely interception of its course. This can facilitate the initiation and/or enhancement of evidence-based treatments early on, ultimately leading to improved long-term outcomes for patients with HFmrEF.

FMR is secondary to left heart structure and dysfunction, making drugs that improve ventricular remodeling the preferred treatment for FMR. This includes ACEI or ARBs, β-blockers, and mineralocorticoid receptor antagonists. Recent studies have shown that ARNI and sodium-glucose symporter 2 inhibitors in GDMT can also improve FMR in patients with HFmrEF [22, 23]. With the advent of interventional techniques, new treatment options have emerged for FMR. COAPT [24] and MITRA-FR [25] examined the outcomes of patients with FMR who underwent transcatheter mitral-valve repair (TMVr) and observed their long-term prognosis. The findings presented conflicting conclusions, leading to debate over the efficacy of interventional therapy for patients with FMR. This also prompts the question of identifying the specific criteria for determining which patients with FMR would benefit from interventional treatment. Crayburn et al. were the first to suggest that TMVr may only benefit patients with FMR when their degree of FMR is disproportionate to left ventricular remodeling [26]. In a retrospective analysis, they compared those with proportional and disproportionate left ventricular remodeling who underwent TMVr. The study found that only FMR patients with disproportionate left ventricular remodeling showed reverse left ventricular remodeling [27]. In this study, 4 patients underwent TMvr. In addition to anatomical criteria for FMR, other considerations include HF symptoms persisting despite standardized drug treatment, severe FMR disproportionate to left ventricular remodeling, LVEF between 20% and 50%, LVESD ≤ 70 mm, pulmonary artery systolic pressure ≤ 70 mmHg, absence of right ventricular dysfunction, and absence of severe tricuspid regurgitation.

### Limitations

Firstly, this was a single-center study with a small sample size. Further multi-center cohort studies will still be needed to investigate the relationship between FMR and HFmrEF prognosis. Secondly, as in other epidemiological studies, [11, 21, 28] echocardiography did not assess FMR changes at discharge and during follow-up, which could have influenced our observations. Finally, the study did not explore the mechanisms of mitral regurgitation, and the association between the etiology of HF and the mechanism of FMR remains to be determined. Real-time three-dimensional ultrasound offers a more precise pathological and morphological basis for diagnosing and assessing FMR, addressing the limitations of two-dimensional ultrasound in accurately determining the regurgitant jet’s shape, direction, and size. Future research will incorporate 3D echocardiography for evaluating FMR and analyzing its impact on HF prognosis.

## Conclusions

The findings of this study revealed a heightened occurrence of moderate and moderate-severe/severe FMR among patients diagnosed with HFmrEF. Furthermore, a notable correlation was observed between moderate and moderate-severe/severe FMR and a significantly elevated risk of unfavorable outcomes in HFmrEF patients, establishing FMR as an independent risk factor impacting clinical prognosis. Given these results, future therapeutic approaches for HFmrEF patients may focus on innovative strategies to ameliorate FMR, thereby seeking improvements in the overall management of this condition.

## Data Availability

The data that support the findings of this study are not openly available due to reasons of sensitivity and are available from the corresponding author upon reasonable request. Data are located in controlled access data storage at the Second Affiliated Hospital of Shenzhen University.

## Abbreviations

FMR: Functional mitral regurgitation
HF: Heart failure
HFmrEF: Heart failure and mildly reduced ejection fraction
HFrEF: Heart failure with reduced ejection fraction
HFpEF: Heart failure with preserved ejection fraction
LVEF: Left ventricular ejection fraction
AHF: Acute heart failure
GDMT: Guideline-directed medical therapy
LVEDD: Left ventricular end-diastolic diameter
LVESD: Left ventricular end-systolic diameter
LAD: Left atrial diameter
RVSP: Right ventricular systolic pressure
MVEmax: Mitral value maximum E velocity
HR: Hazard ratio
ESC: European Society of Cardiology
NYHA: New York Heart Association
NT-pro-BNP: N-terminal-B-type natriuretic peptide
MVR: Mitral valve repair
ACEI: Angiotensin-converting enzyme inhibitor
ARB: Angiotensin receptor blocker
ARNI: Angiotensin receptor neprilysin inhibitor
BMI: Body mass index
